# Relationship between Obesity and Massive Transfusion Needs in Trauma Patients, and Validation of TASH Score in Obese Population: A Retrospective Study on 910 Trauma Patients

**DOI:** 10.1371/journal.pone.0152109

**Published:** 2016-03-24

**Authors:** Audrey De Jong, Pauline Deras, Orianne Martinez, Pascal Latry, Samir Jaber, Xavier Capdevila, Jonathan Charbit

**Affiliations:** 1 Trauma Intensive Care & Critical Care Unit, Lapeyronie University Hospital, Montpellier, France; 2 Intensive Care Unit & Anesthesiology Department, Saint-Eloi University Hospital, Montpellier, France; 3 INSERM U1046 Montpellier, France; King Abdullah International Medical Research Center, SAUDI ARABIA

## Abstract

**Background:**

Prediction of massive transfusion (MT) is challenging in management of trauma patients. However, MT and its prediction were poorly studied in obese patients. The main objective was to assess the relationship between obesity and MT needs in trauma patients. The secondary objectives were to validate the Trauma Associated Severe Hemorrhage (TASH) score in predicting MT in obese patients and to use a grey zone approach to optimize its ability to predict MT.

**Methods and Findings:**

An observational retrospective study was conducted in a Level I Regional Trauma Center Trauma in obese and non-obese patients. MT was defined as ≥10U of packed red blood cells in the first 24h and obesity as a BMI≥30kg/m². Between January 2008 and December 2012, 119 obese and 791 non-obese trauma patients were included. The rate of MT was 10% (94/910) in the whole population. The MT rate tended to be higher in obese patients than in non-obese patients: 15% (18/119, 95%CI 9‒23%) versus 10% (76/791, 95%CI 8‒12%), OR, 1.68 [95%CI 0.97‒2.92], *p* = 0.07. After adjusting for Injury Severity Score (ISS), obesity was significantly associated with MT rate (OR, 1.79[95%CI 1.00‒3.21], *p* = 0.049). The TASH score was higher in the obese group than in the non-obese group: 7(4–11) versus 5(2–10)(*p*<0.001). The area under the ROC curves of the TASH score in predicting MT was very high and comparable between the obese and non-obese groups: 0.93 (95%CI, 0.89‒0.98) and 0.94 (95%CI, 0.92‒0.96), respectively (*p* = 0.80). The grey zone ranged respectively from 10 to 13 and from 9 to 12 in obese and non obese patients, and allowed separating patients at low, intermediate or high risk of MT using the TASH score.

**Conclusions:**

Obesity was associated with a higher rate of MT in trauma patients. The predictive performance of the TASH score and the grey zones were robust and comparable between obese and non-obese patients.

## Introduction

The early detection of patients at risk of massive transfusion (MT) is nowadays the main challenge of initial management of bleeding trauma patients. The early and aggressive triggering of haemostatic therapy was indeed associated with an improved outcome in these patients [1, 2]. Some scores such as the Trauma Associated Severe Hemorrhage (TASH) score [3, 4] were developed for this specific purpose and validated for predicting MT requirements as soon as admission of trauma patients. The TASH score has recently been proved to have the best predictive performance [4]. Most experts encourage prediction of massive bleeding to guide initial resuscitation using this kind of composite scoring system [1].

Meanwhile, obesity has become a worldwide health concern [5]. Similarly in trauma, mortality and morbidity have been shown to be higher in severely injured obese patients compared with non-obese patients (6–9). A recent report revealed that obese trauma patients were at higher risk of mortality in the initial phase after trauma, mainly due to persistent hemorrhagic shock [6]. Some mechanisms that might explain this increased mortality were identified in obese traumatized rats with a hemodynamic instability; a K-ATP channel-dependent decrease in arterial tone would be responsible for alterations in the vasoconstriction response, worsening the shock state [7]. Furthermore, an experimental study showed that the positive inotropic effect of β-adrenoceptor stimulation was slightly decreased in obese Zucker rats [8].

Many other clinical arguments could also explain higher propensity to blood spoliation following trauma in obese patients. Some clinical or ultrasonographic diagnoses may for example be more difficult to establish in obese patients, which may delay the required management [9–11]. Similarly, interventional procedures such as central venous catheters [12], intubation [13, 14], mechanical ventilation [15], orthopedic stabilization [16], or thoracic and abdominal surgery are reputed to be more difficult in obese patients than in non-obese patients [17]. For these reasons, obese trauma patients should be considered as a specific population, particularly in terms of transfusion risk. However, to our knowledge, the risk of MT has never been specifically assessed depending on obesity. Moreover, although the TASH score is well known to be a robust scoring system for predicting MT requirements, its predictive performance has never been validated in obese patients. Specific thresholds therefore need to be determined in this specific population.

The main aim of this study was to compare MT needs according to obesity status in a population of trauma patients. Our hypothesis was that obesity was associated with increased MT needs. The secondary aims were to compare the predictive performance of TASH score for MT between obese and non obese patients and to provide clinical relevant thresholds using a grey zone approach in these two populations.

## Materials and Methods

### Design and setting

We carried out a retrospective review of all trauma patients admitted consecutively to the intensive care unit (ICU) of the Level I Regional Trauma Center of Lapeyronie University Hospital between January 2008 and December 2012. Based on the French guidelines for prehospital medical triage, all patients living in the city of Montpellier (France) and its region suspected to have sustained a severe trauma are directly transferred from scene by prehospital team to this unit and managed following a standardized work-up [18].

All trauma patients managed in this trauma unit during this period were included (n = 910). The following were excluded: (1) patients who died immediately after admission (defined as patients who have died within an hour following admission, n = 18); (2) patients not directly admitted to our hospital (n = 93); (3) patients with important clinical data missing in the hospital database (n = 72). Because of its retrospective and observational nature, the need for written consent for this study was not required by our institutional ethical committee (name of the ethics committee which approved the study: COGAR: "Comité d'Organisation et de Gestion de l'Anesthésie Réanimation"). Patient information was anonymized and de-identified prior to analysis. Almost the half of these patients has been included in previous retrospective studies on MT [19, 20].

### Data collection

Data extracted from medical records comprised detailed information on demographics, anthropometrics, clinical and biological data on hospital admission, as well as the Simplified Acute Physiology Score II (SAPS II) [21], the Abbreviated Injury Scale for each region (AIS) [22] and the Injury Severity Score (ISS) [23]. Transfusion needs were extracted from blood bank records.

### Determination of TASH score

The following variables needed to calculate the TASH score were collected [3, 24]: systolic arterial blood pressure and heart rate on admission to the emergency department, hemoglobin and base deficit on admission samples, complex pelvic fracture (defined as Tile B and C fractures), open or complex femur fracture. The TASH score was then calculated *a posteriori* using standard rules (Table 1) [3].

**Table 1:** TASH score.

Variable	Value	Points
Hemoglobin (g/dL)	<7	8
	<9	6
	<10	4
	<11	3
	<12	2
Base excess (mM)	<−10	4
	<−6	3
	<−2	1
Systolic blood pressure (mmHg)	<100	4
	<120	1
Heart rate (bpm)	>120	2
Free intraabdominal fluid		3
Clinically instable pelvic fracture		6
Open or dislocated femur fracture		3
Male gender		1

### Definition of Obesity classes

Obesity was assessed as a four-category variable according to the World Health Organization (WHO) classification [25]: underweight, body mass index (BMI) < 18.5 kg/m^2^; normal weight, 18.5 kg/m^2^ ≤ BMI < 25 kg/m^2^; overweight, 25 kg/m^2^ ≤ BMI < 30 kg/m^2^; and obese, BMI ≥ 30 kg/m^2^). From this analysis, two groups were determined: obese (BMI ≥ 30 kg/m^2^) and non-obese (BMI < 30 kg/m^2^).

### Outcome measurements

The primary end point was the rate of MT, defined as the administration of 10 units of packed red blood cells within the 24 first hours or death due to hemorrhagic shock [19]. During study period, transfusion strategy and triggering criteria of MT were not protocolized and were left to the physician appreciation.

Mortality in the ICU and in the hospital, length of ICU stay (excluding the patients who died in the first week) and length of hospital stay (excluding the patients who died in the first week) were secondary end points. The 28-day ICU free days were calculated by subtracting the actual ICU length of stay in days from 28. The 28-day ICU free day was considered 0 if a patient died before hospital discharge or stayed in the ICU for more than 28 days.

### Statistical analysis

Quantitative variables were expressed as means (standard deviation [SD]) or medians (interquartile range 25%–75%) and compared between obese and non obese patients using the Student *t*-test or the Wilcoxon test as appropriate (Gaussian or non-Gaussian variables). Qualitative variables were expressed as numbers (percentage) and compared using the chi-square test or the Fisher test as appropriate. The comparison of MT rates was *a priori* planned between obese and non obese groups and also according to the level of obesity using the Mantel-Haenzel test [26]. These percentages were provided with their 95% confidence intervals (CI). Logistic regression was performed to assess the strength of the relationship between obesity and MT, before and after adjusting for ISS score. Obesity status was assessed in this analysis as a binary variable and as a multi-class variable using the WHO classification. Correlation of TASH score and ISS was assessed using the Pearson or Spearman correlation according to distribution of variables.

Received operating characteristic (ROC) curve analysis was secondarily performed to assess the ability of the TASH score to predict MT in the obese and non-obese groups. The areas under the ROC curves (AUCs) were provided with their 95% CIs and compared according to the Hanley and McNeil method [27]. The goodness of fit of the scoring system was assessed by the Hosmer-Lemeshow test [28]. Thereafter, thresholds analysis was carried out from the ROC curves using the grey zone method, assuming that a simple threshold (e.g. the Youden method) cannot both predict and exclude a risk. The grey zone corresponds to a range of values for which formal conclusions cannot be obtained. After determining the values for this interval, three classes of response were defined: low, intermediate, and high risk. This inconclusive grey zone was first defined by the standard method: the high boundary was determined as the highest threshold with a positive likelihood ratio (LR) lower than 10, while the low boundary was the lowest threshold with a negative LR higher than 0.1 [29]. For each threshold, sensitivity, specificity, predictive values and LRs are provided. In order to better present these results, a two-curve (sensitivity, specificity) representation was also built. This standard approach was completed with an alternative approach described by Canesson et al. [30]. After assessing the “optimal” threshold using Youden’s index (*J* = sensitivity + specificity − 1), Youden’s index was determined for 1000 bootstrapped populations, resulting in a set of 1000 “optimal” values. The mean value of these optimal values and its 95% CI were then estimated; this 95% CI defined an alternative approach to the grey zone. The three classes of response obtained after determining the grey zone were compared in each group in terms of MT requirements, death rate and duration of hospitalization.

Statistical significance was considered at *p* < 0.05 and *p* values were two-tailed. The statistical analysis was performed by the Medical Statistical Department of the Montpellier University Hospital with the help of statistical software (SAS, version 9.3; SAS Institute; Cary, NC and R, version 2.14.1 and Medcalc, version 14.8.1).

## Results

### General population

**Fig 1:**
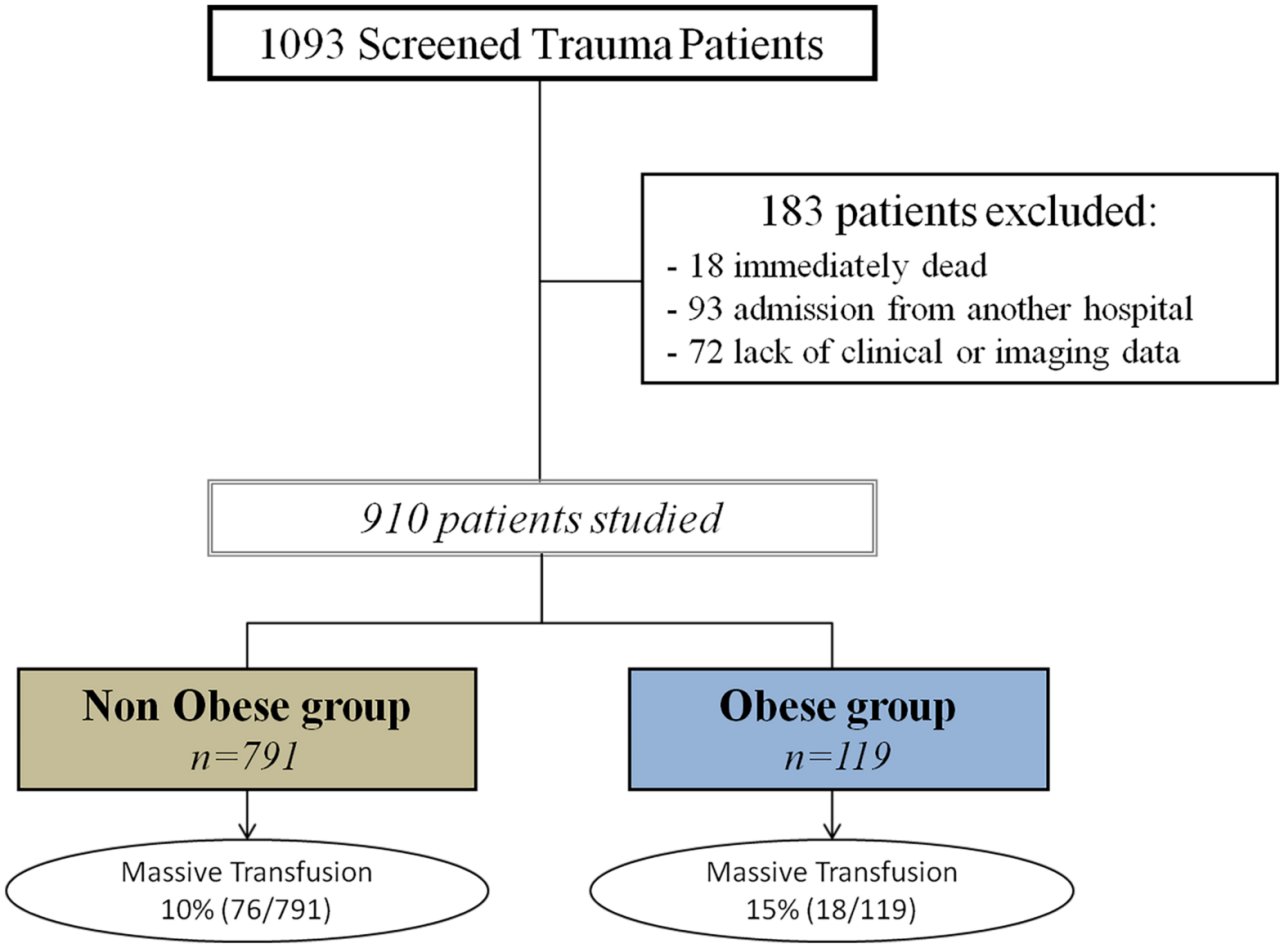
Patient flow diagram.

During the 5-year study period, 1093 patients were admitted to our ICU for trauma and 183 (17%) met the exclusion criteria. The 910 remaining patients (83%) were analyzed in the present study; the details of the exclusion process are shown in Fig 1. The mean age of the population was 40±19 years and the mean ISS was 19±13. The cause of trauma was as follows: 556 motor vehicle crashes, 35 pedestrians struck by a motor vehicle, 107 bicycle crashes, 140 falls and 72 assaults. The overall MT rate was 10% (94/910; 95% CI 8‒12%) and the death rate was 4% (39/910). In this population, 119 patients (13%) were classified as obese and 791 (87%) were non-obese (469 [51%] normal weight, 280 [31%] overweight, 42 [5%] underweight). Table 2 presents the main characteristics of the patients according to their BMI classes. TASH score and ISS were significantly correlated (*R* = 0.44, *p* <0.001).

**Table 2:** Baseline population characteristics in obese and non-obese patients.

Variables	All patients (*n* = 910)	Obese (*n* = 119)	Non-obese (*n* = 791)	p-value
Age (yr), median (interquartile range)	37 (23–53)	49 (37–64)	34 (23–50)	<0.001
Gender (% male)	697 (77)	89 (75)	608 (77)	0.62
Weight (kg), median (interquartile range)	73 (65–84)	98 (90–109)	70 (62–80)	<0.001
Height (cm), median (interquartile range)	174 (166–180)	174 (165–180)	174 (166–180)	0.42
BMI (kg/m²), median (interquartile range)	24 (22–27)	32 (31–35)	24 (21–26)	<0.001
Mechanism of injury				0.36
Motor vehicle crash (%)	556 (61)	73 (61)	483 (61)	
Pedestrian (%)	35 (4)	7 (6)	28 (4)	
Bicycle (%)	107 (12)	10 (8)	97 (12)	
Fall (%)	140 (15)	22 (16)	118 (15)	
Assault (%)	72 (8)	7 (6)	65 (8)	
ER SBP < 90 mmHg (%)	331 (36)	56 (47)	275 (35)	0.01
BD ≥ 6 mmol/L (%)	215 (24)	34 (29)	181 (23)	0.17
Severe anatomic injuries				
Head AIS ≥ 3 (%)	231 (26)	27 (23)	204 (26)	0.52
Chest AIS ≥ 3 (%)	261 (29)	32 (27)	229 (29)	0.68
Abdominal AIS ≥ 3 (%)	208 (23)	27 (23)	181 (23)	0.96
Extremity AIS ≥ 3 (%)	246 (27)	46 (39)	200 (25)	0.002
ISS, median (interquartile range)	17 (10–26)	17 (13–25)	17 (9–26)	0.55
ISS ≥ 16 (%)	509 (56)	69 (58)	440 (56)	0.56
SAPS II score, median (interquartile range)	26 (17–37)	31 (21–39)	25 (15–37)	0.002
Hemoglobine (g/dL), median (interquartile range)	12.0 (9.8–13.7)	11.8 (10.6–13.4)	12.0 (9.8–13.8)	0.81
TASH score, median (interquartile range)	5 (2–10)	7 (4–11)	5 (2–10)	<0.001
28-day ICU free days, median (interquartile range)	20 (8–24)	14 (0–22)	20 (9–24)	<0.001
ICU stay^a^, median (interquartile range)	8 (4–18)	12 (5–27)	8 (4–17)	<0.001
Hospital stay^a^, median (interquartile range)	18 (11–32)	24 (14–40)	17 (10–31)	<0.001
ICU mortality, median (interquartile range)	39 (4)	8 (7)	31 (4)	0.16

AAST = American Association for the Surgery of Trauma, AIS = Abbreviated Injury Scale, BD = base deficit, ER SBP = emergency room systolic arterial blood pressure, FFP = fresh frozen plasma, ICU = intensive care unit, ISS = Injury Severity Score, TASH = trauma associated severity score, MVC = motor vehicle crash, PRBCs = packed red blood cells, SAPS = Simplified Acute Physiology Score.

^a^Excluding the patients who died in the first week.

### Obesity and MT requirements

**Fig 2:**
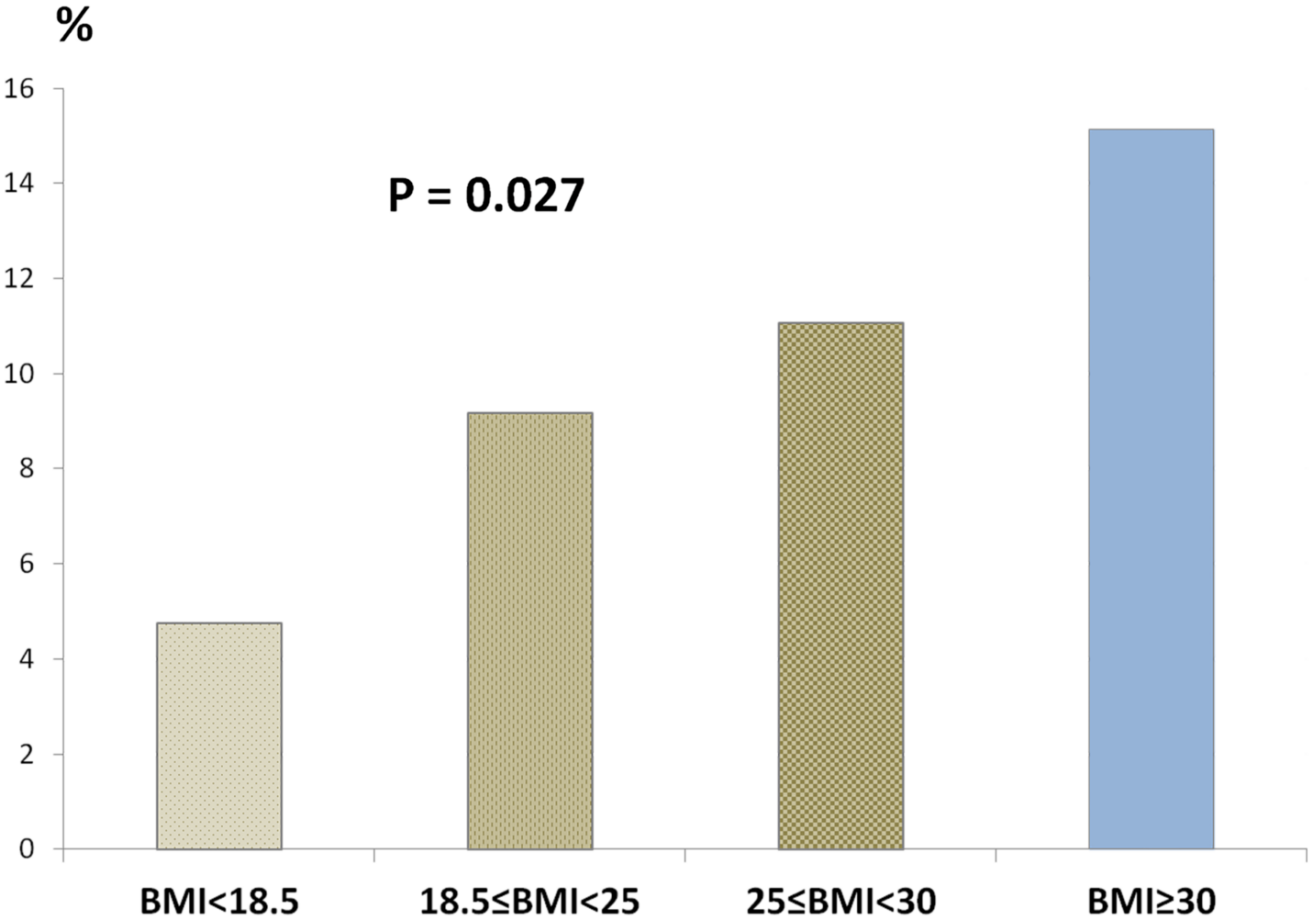
Rates of massive transfusion according to the level of obesity. Rates of massive transfusion were significantly higher with an increased BMI (*p* = 0.027).

The MT rate tended to be higher in obese patients than in non-obese patients: 15% (18/119; 95% CI 9‒23%) versus 10% (76/791; 95% CI 8‒12%), *p* = 0.07. The OR of the association between obesity and MT was 1.68 [95% CI 0.97‒2.92]. After adjusting for ISS score, this association was significant: adjusted OR, 1.79 [95% CI 1.00‒3.21], *p* = 0.049. Using the WHO classification, an increased BMI was also significantly associated with a higher MT rate (Fig 2): 5% (2/42; 95% CI 1‒16%) in underweight, 9% (43/469; 95% CI 7‒12%) in normal weight, 11% (31/280; 95% CI 8‒15%) in overweight and 15% (18/119; 95% CI 9‒23%) in obese patients (*p* = 0.027).

### TASH score and MT prediction

**Fig 3:**
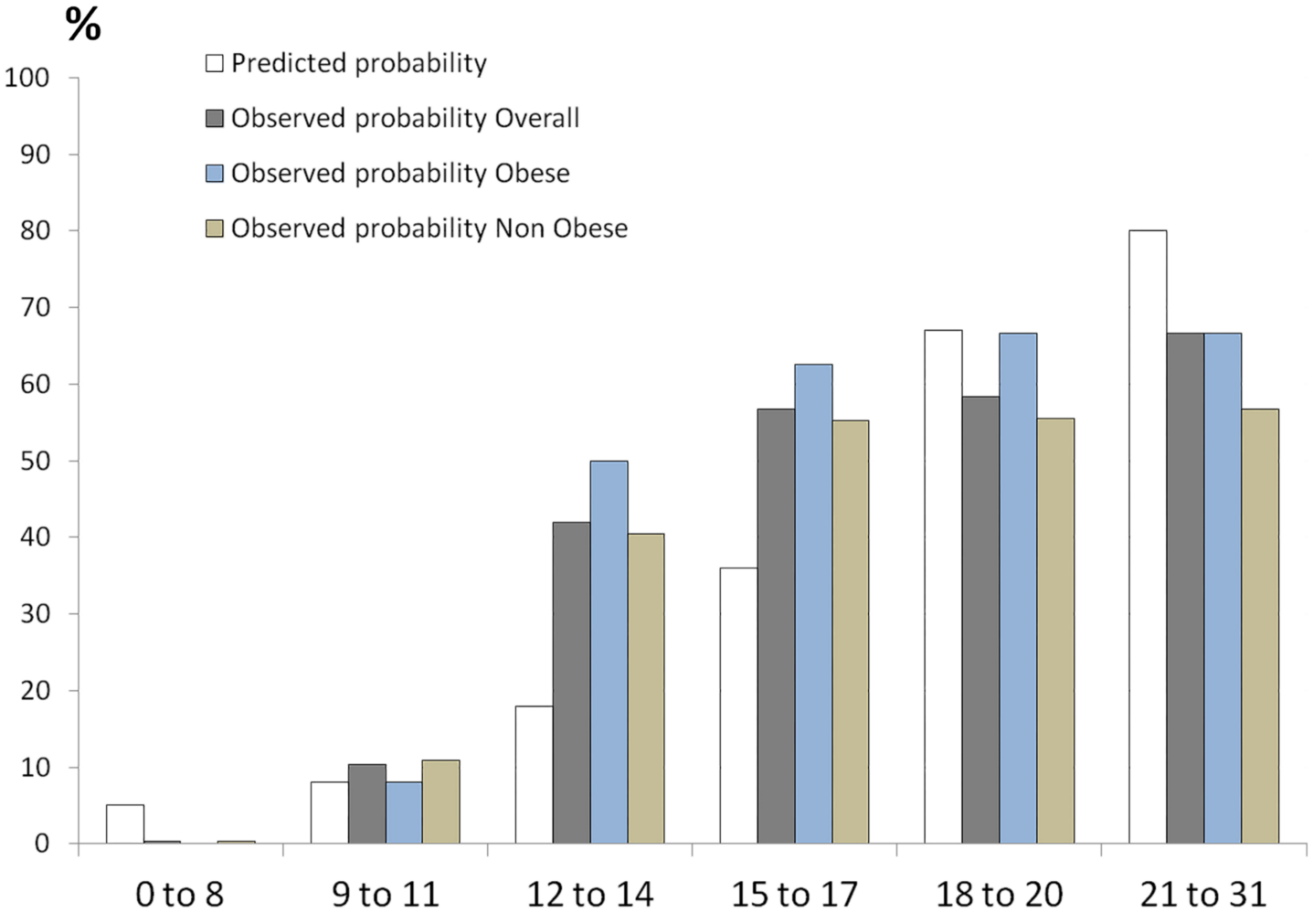
Predicted and observed probability for the TASH score. This figure presents the predicted probability of MT for the TASH score determined in the study of Maegele et al. [31]. The observed probabilities of MT for the TASH score in the present study are shown in the overall population, obese patient and non-obese patients. The higher the TASH score was, the more frequently MT was required.

**Fig 4:**
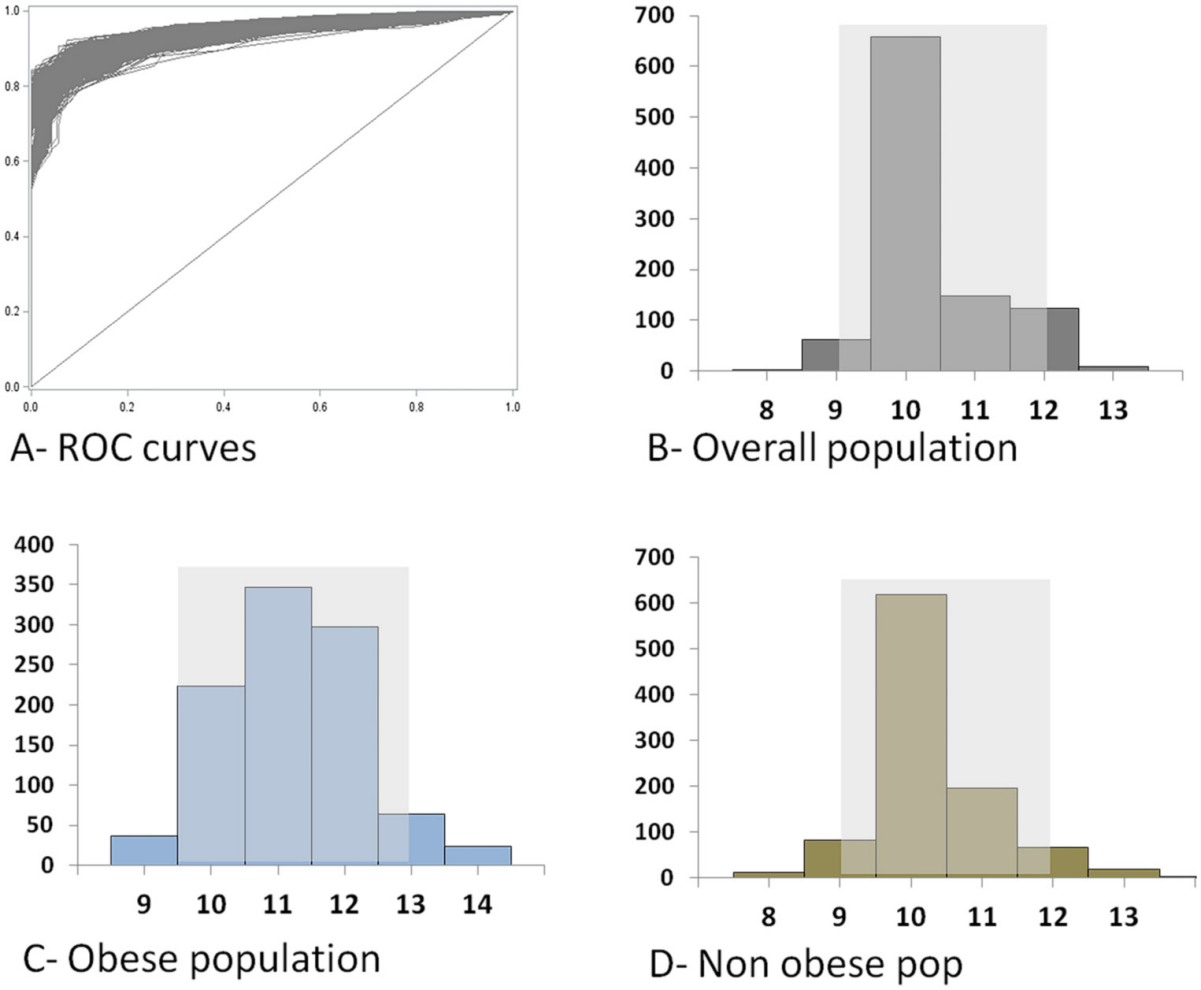
ROC curve analysis for the TASH score in predicting MT. (A) ROC curves for each population (grey, general population; blue, obese population; maroon, non-obese population). (B–D) Determination of the standard approach for the grey zone using the likelihood ratio method. Sensitivity and specificity of the TASH score according to the value of the cutoff. The inconclusive grey zone has been represented as a grey rectangle: 9–12 for the overall population (B), 10–13 in the obese population (C) and 9–12 the non-obese population (C).

**Fig 5:**
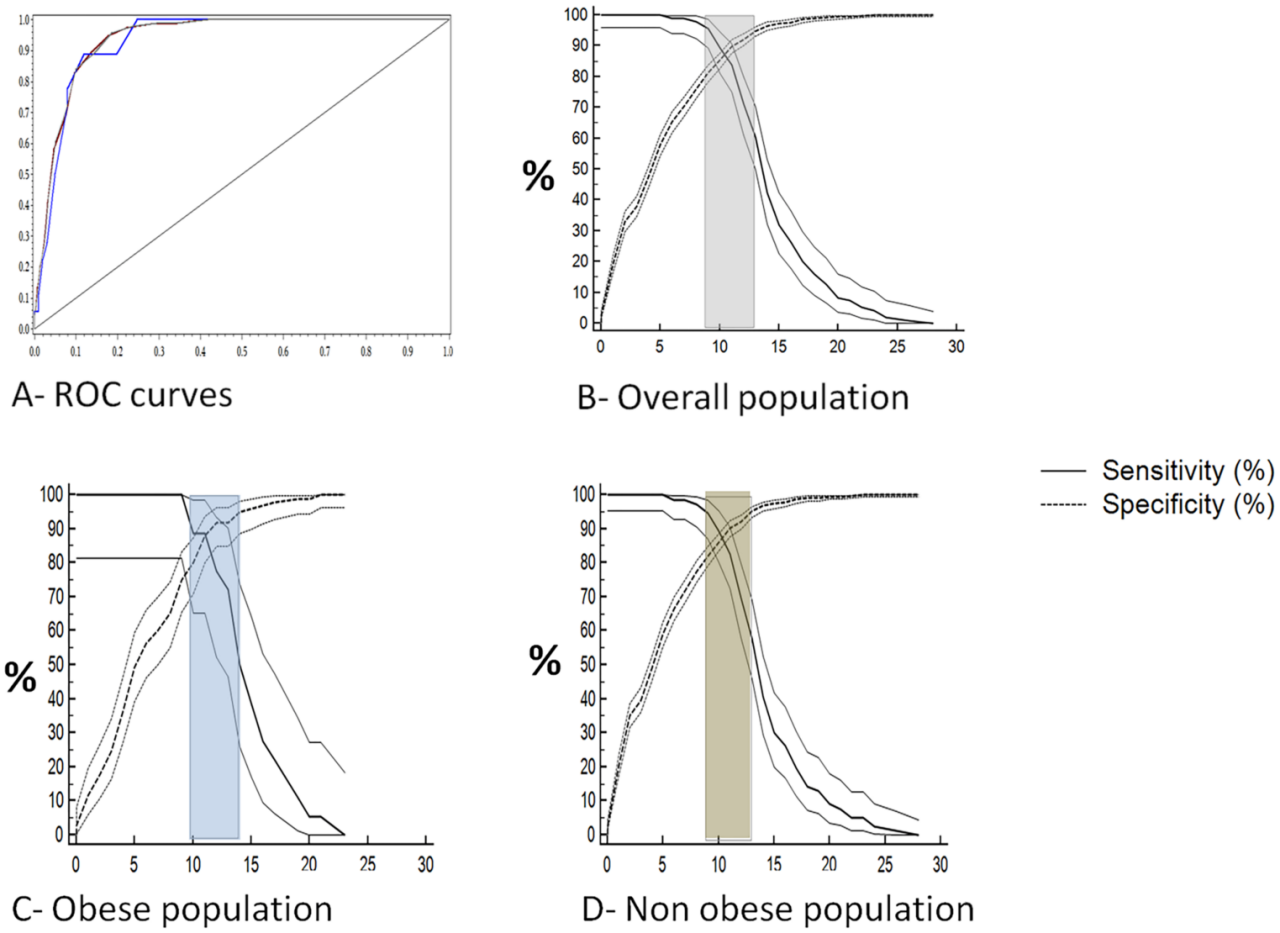
Bootstrapping analysis and the alternative approach. (A) ROC curves bootstrapped. Only the bootstrap of the ROC curve for the overall population is shown on this figure. (B–D) Determination of the alternative approach for the grey zone using the bootstrapping method: 9–12 for the overall population (B), 9.5–13 in the obese population (C) and 9–12 in the non-obese population (D).

The median TASH score was 5 (2‒10) in the overall population. The higher the TASH score, the more frequently MT was required (Fig 3). The global ability of the TASH score to predict MT in the overall population was provided by ROC curve analysis, with an AUC of 0.94 (95% CI 0.92‒0.95). The *p* value of the goodness of fit of the TASH score (Hosmer and Lemeshow test) was 0.43. Using the standard, the inconclusive grey zone was defined from 9 to 12 (Fig 4). The statistical performances of the border thresholds are detailed in Table 2. Nine percent of patients (84/910) were included in this inconclusive zone. Using an alternative approach, the grey zone was similar, ranging from 9 to 12 (Fig 5).

### TASH score and obesity

The TASH score was slightly higher in the obese group than in the non-obese group: 7 (4–11) versus 5 (2–10) (*p* < 0.001). ROC analysis showed that the AUC of the TASH score for predicting MT was comparable in the obese and non-obese groups; 0.93 (95% CI 0.89‒0.98) and 0.94 (95% CI 0.92‒0.96), respectively (*p* = 0.80). The corresponding *p* values of the goodness of fit of the TASH score were 0.73 and 0.35, respectively. The grey zone determined by the standard approach ranged from 10 to 13 and from 9 to 12, respectively (Fig 4). The statistical performances of the border thresholds in the obese and non-obese populations are presented in Table 3. Respectively 13% (15/119) and 8% (67/791) of patients were included in the inconclusive grey zone (*p* = 0.14). Alternative approach of grey zones found almost identical findings: 9.5‒13 and 9‒12, respectively (Fig 5).

**Table 3:** Ability of the TASH score to predict MT in obese and non-obese patients.

Variables	All patients (*n* = 910)	Obese (*n* = 119)	Non-obese (*n* = 791)	
**Minimal threshold**	**≥9**	**≥10**	**≥9**	
Se (%)	96 (90–99)	90 (65–99)	95 (87–99)	0.03
Sp (%)	81 (79–84)	80 (71–88)	82 (79–85)	0.56
PPV (%)	37 (31–44)	44 (28–62)	36 (30–43)	0.11
NPV (%)	99 (99–100)	98 (92–100)	99 (98–100)	0.51
+LR	5 (4–6)	4 (3–7)	5 (5–6)	0.16
−LR	0.05 (0.02–0.1)	0.1 (0.04–0.5)	0.06 (0.02–0.2)	0.89
**Optimal threshold**	**≥10**	**≥11**	**≥10**	
Se (%)	89 (81–95)	90 (65–99)	89 (80–95)	0.56
Sp (%)	86 (83–88)	88 (80–94)	86 (84–89)	0.50
PPV (%)	42 (35–49)	57 (37–76)	41 (33–49)	0.70
NPV (%)	99 (97–99)	98 (92–100)	99 (98–99)	0.51
+LR	6 (5–7)	8 (4–13)	7 (5–8)	0.68
−LR	0.12 (0.07–0.2)	0.1 (0.03–0.5)	0.12 (0.06–0.2)	0.24
**Maximal threshold**	**≥13**	**≥14**	**≥13**	
Se (%)	61 (50–71)	50 (27–74)	58 (46–69)	0.12
Sp (%)	95 (93–96)	95 (89–98)	95 (94–97)	1.00
PPV (%)	58 (48–68)	64 (35–87)	56 (40–72)	0.11
NPV (%)	95 (94–97)	91 (84–96)	93 (91–95)	0.40
+LR	12 (9–17)	10 (4–27)	13 (9–18)	0.24
−LR	0.4 (0.3–0.5)	0.5 (0.3–0.8)	0.8 (0.7–0.9)	0.007

Values are expressed with their 95% CI in parentheses. −LR = negative likelihood ratio, +LR = positive likelihood ratio, MT = massive transfusion, NPV = negative predictive value, PPV = positive predictive value, Se = sensitivity, Sp = specificity, TASH = Trauma Associated Severe Hemorrhage.

### TASH score stratification and outcome

**Fig 6:**
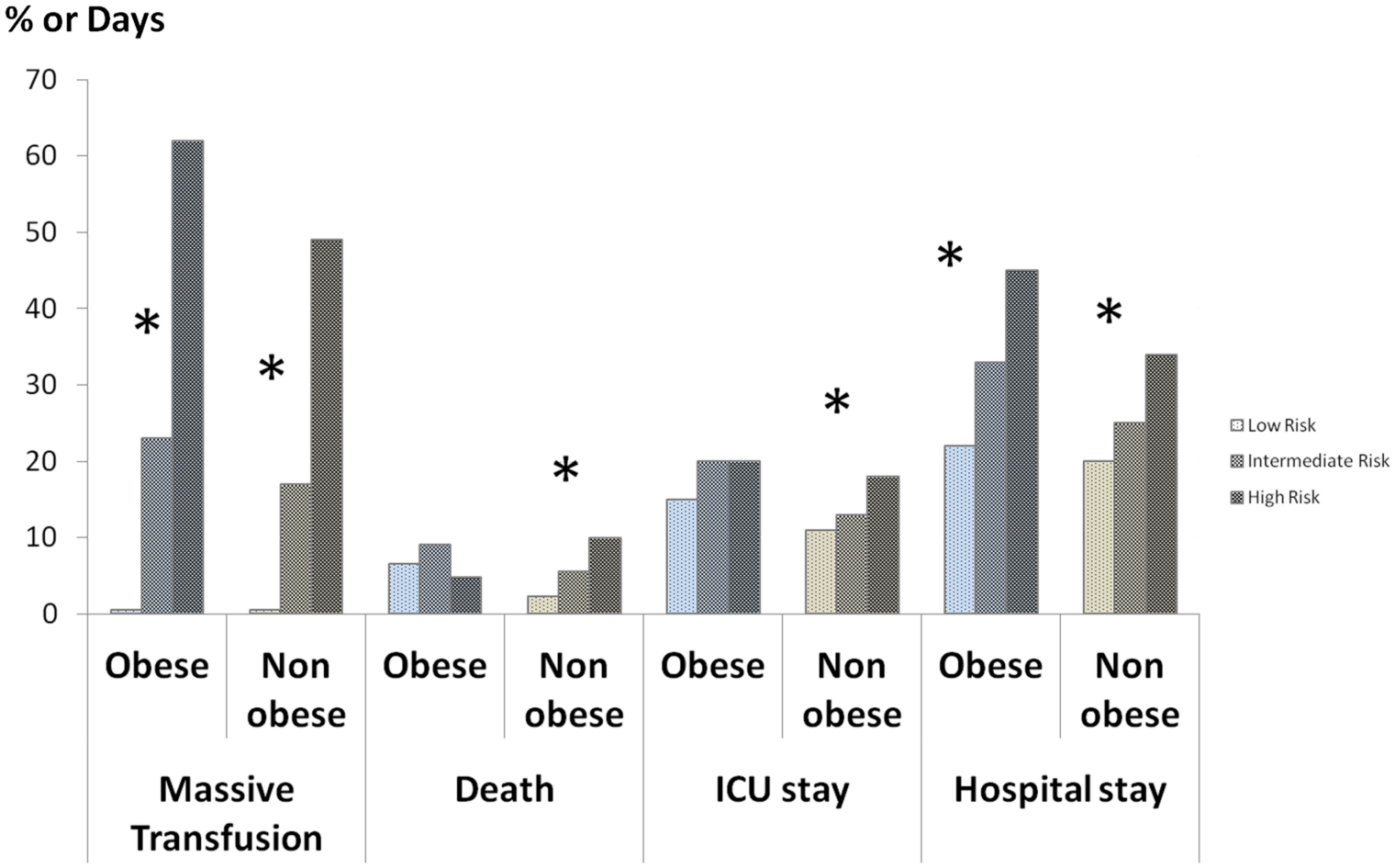
Rates of massive transfusion, death, length of ICU and hospital stays according to the TASH score in the obese and non-obese groups. * *p* < 0.05. The risk subgroups were significantly associated with MT likelihood and length of stay in hospital in the obese and non-obese populations (*p* < 0.05). These subgroups were also associated with the death rate and length of stay in the ICU in the non-obese population (*p* < 0.05).

According to the respective grey zones obtained by the standard approach, obese and non-obese populations were separated into three subgroups using the TASH score: low, intermediate and high risk. These risk subgroups were significantly associated with MT likelihood (respectively of 0.5%, 23% and 62% in the obese; 0.5%, 17% and 49% non obese) and length of stay in hospital (respectively of 22, 33 and 45 days in the obese; 20, 25 and 34 days in non-obese) in the obese and non-obese populations (*p* < 0.05). These subgroups were also associated with the death rate (respectively of 7%, 9% and 5% in obese; 2%, 6% and 10% in non-obese) and length of stay (respectively of 15, 20 and 20 days in the obese; 11, 13 and 18 days in non-obese) in the ICU in the non-obese population (*p* < 0.05) (Fig 6).

## Discussion

In this 5-year study performed on 910 trauma patients, we found that obesity was significantly associated with MT needs, when considering trauma severity. Furthermore, higher BMI classes, defined by the WHO classification, were associated with an increased risk of MT. In the present series, we validated this association for the first time in an identifiable population of obese trauma patients. The global ability of the TASH score to predict MT was robust and comparable between the obese population and the non-obese population (AUC 0.93–0.94). The determination of inconclusive grey zones for the TASH score was also an important aim of our analysis. We observed almost similar inconclusive zones in obese and non-obese patients, 10–13 and 9–12, respectively. The current study highlighted the relevance of the grey zone approach for the TASH score because the classification into three risk subgroups (low, intermediate and high risk) was associated with all the outcome variables (MT likelihood, death rate, ICU stay and hospital stay) in non-obese patients, and with MT likelihood and hospital stay in obese patients.

Worldwide, the incidence of obesity varies from less than 10% to more than 30%, depending on the sex and the country [32]. In France, this rate tends to be low with 11% of the population classified as obese in 2003 [33]. After multiple controversies [34], obesity seems to be associated with decreased complications, morbidity and mortality in critically ill patients [35–37]. In contrast, obesity has been associated with a worse outcome in trauma patients [38–42]. In the present cohort, obese and non-obese populations were comparable in terms of injury severity, excepting for severe extremity injuries that were slightly more numerous in obese group (Table 1). Our findings have demonstrated that BMI classes were associated with MT needs. This significant relationship was especially observed after adjusting for ISS value. To explain this increased risk of bleeding in obese patients, many causes may be put forward. First, these patients are more difficult to assess in the early phase. Clinical diagnosis of obese patients is indeed more challenging; in the same way, ultrasound examination is known to be less reliable due to altered contrast [10, 11]. Thus, several diagnostic or clinical pitfalls may be seen too late, which will delay the start of haemostatic therapy, worsening the shock state and the prognosis. Second, resuscitation procedures such as intravenous access or arterial line placement are reputedly more difficult to perform [12, 17]. This may also delay administration of the appropriate strategy. Third, cutaneous lacerations and wounds may lead to larger blood loss from abundant and hyper-vascularized adipose tissue. Fourth, obese patients might be more likely to have severe or complex fractures, as found in our series (Table 1). Fifth, many surgical procedures are challenging due to more difficult and prolonged intervention, poor visibility, requiring longer instruments and additional assistants [16]. Finally, hemodynamic responses and associated hormone secretion were found to be impaired in an obese experimental model [7], as the positive inotropic effect of β-adrenoceptor stimulation [8]. Physiological disturbances can aggravate shock and increase the occurrence of multiple organ failure or trauma-induced coagulopathy. It is thus interesting to note in our series that rate of hypotensive patients in obese group was higher than non-obese one (Table 1).

The TASH scoring system was built to predict MT using admission parameters such as mean occult shock, blood spoliation or specific bleeding injuries. This score was found to have the highest overall accuracy in comparison with other composite scoring systems established for predicting MT, with AUCs included between 0.89 and 0.91 in most studies [4]. In the present study, the AUC found was slightly higher in our overall population (0.94). One explanation could be that, over the last 10 years, clinicians in our center have become accustomed to use the TASH score at admission to trigger aggressive transfusion [3, 4, 31]. The present series is the first study to assess and validate the performance of the TASH score in a specific population of obese patients. Despite the difference in terms of transfusion requirements, the AUC for the obese patients in our analysis was comparable with the AUC for the non-obese patients (0.93 versus 0.94). Similarly, observed probabilities of different values of the TASH score were comparable between the two groups (Fig 3). Our findings underscore that criteria of the TASH score remain strongly predictive of massive blood loss even in an obese population more at risk of MT and that the statistical performance of the TASH score is excellent in an obese trauma population.

Previous series on the TASH score have already provided several statistical thresholds; the optimal threshold for Yucel et al. [3] was 16, whereas it was 8.5 for Brockamp et al. [4]. This large gap can be explain by the different ways of assessing the optimal thresholds, using either the Youden's index (sensitivity + specificity − 1) or the point on the ROC curve closest to (0;1), respectively [43]. Statistical debate remains about the best criterion for classification accuracy [44]. Youden's index reflects mathematically the intention of maximizing the overall correct classification rates and thus minimizing misclassification rates, while the point on the ROC curve closest to (0,1) involves a quadratic term for which the clinical meaning is unknown. Anyway, the use of an optimal threshold remains limited and leads to a binary constraint of a “black or white” decision, which is often inappropriate in clinical or screening practice [45]. A unique cutoff value does not allow the test results to simultaneously confirm and exclude diagnostic hypotheses, whereas the grey zone approach offers values that will either confirm or exclude it [46]. This approach is increasingly used in the medical literature [46]. In our analysis, the lower and upper thresholds delimiting the grey zone were 9 and 12, respectively. Interestingly, the number of patients that belonged to this inconclusive zone was very low (9%). When analyzing this grey zone according to obesity status, it can be seen that the boundary thresholds were nearly the same in obese and non-obese populations; 10–13 and 9–12, respectively (Fig 4). The alternative method of bootstrapping Youden's index to determine the grey zone did not change these values (Fig 5). As a result of the grey zone determination, simple thresholds of the TASH score allow physicians to exclude or predict the MT risk with near certainty for most patients, whether obese or not. Below the high sensitive threshold (i.e. <10 in an obese trauma patient or <9 in a non-obese trauma patient), MT likelihood is almost zero, whereas above the high specific threshold (i.e. ≥13 in an obese trauma patient or ≥12 in a non-obese trauma patient), MT risk is major (Table 3). Furthermore, the stratification of the TASH score into three categories using the grey zone in our analysis was significantly associated with increased hospital length of stay in both populations (Fig 6). Therefore, bleeding risk must be considered really carefully in obese trauma patients. For this purpose, TASH score has been proved to be a robust tool to early identify likelihood of massive blood loss and MT risk during initial assessment and to trigger as soon as possible administration of transfusion packs and coagulation factor concentrates. Present study provides thus reliable and specific thresholds for clinical practice, which were comparable in obese and non-obese populations. In addition, our findings might suggest also that some risky strategies should be considered differently in the specific obese population; nonoperative management of high-risk abdominal injuries or early definitive stabilization of complex fractures could for example be associated with a higher bleeding in obese patients. A specific adjustment of therapeutic strategy in these patients would be desirable. Studies assessing appropriate specific strategies for obese population could definitively answer to these questions.

The following limitations should be considered when assessing the clinical relevance of our results. First, this was a retrospective study. However, the amount of missing data was low (72/1093), and the main criteria were collected for all patients: BMI, TASH score and outcome (MT, length of stay in the ICU and in the hospital). Second, the criteria for initiating a transfusion in trauma patients were not standardized and left to the physician appreciation. Getting a massive transfusion is different from needing a massive transfusion, and we cannot exclude an over transfusion in obese patients. However, TASH score was strongly predictive of MT in the specific obese population, with comparable thresholds with the non-obese population, which is an argument against over transfusion in these patients. Third, the low mortality rate in our obese population (8/119) could explain a lack of power in the assessment of the relationship between the TASH score level and risk of death. However, the cohort of obese patients in the current study is very large in comparison with the existing literature and the CI of the AUC was narrow, showing a high predictive ability of MT with the TASH score. Fourth, the prediction of the TASH score in the overall population was slightly higher than in previous studies. However, the difference was minimal and the potential bias induced appears low. Fifth, even if the TASH score appeared to perform best [4], other scores predicting MT such as Assessment of Blood Consumption (ABC) [47], Prince of Wales Hospital (PWH) score [48] or Traumatic Bleeding Severity Score [49] could be assessed in an obese population.

## Conclusions

In conclusion, obesity was associated with an increased risk of MT. In our study, the TASH score was a strong and accurate predictor of MT in both obese and non-obese populations. For the first time, the TASH score was validated in obese patients and a grey zone was established for this predictive score, allowing the MT risk to be affirmed or rejected with accuracy.
